# Revisiting Shimoda's “Shuuchaku-Kishitsu” (Statothymia): A Japanese View of Manic-Depressive Patients

**DOI:** 10.1155/2011/193742

**Published:** 2011-09-15

**Authors:** Hitoshi Tsuda

**Affiliations:** Department of Psychopathology and Psychotherapy, Nagoya University, Furo-cho, Chikusa-ku, Nagoya-shi 464-8601, Japan

## Abstract

Although the empiric paradigm is now dominant in academic research, in Japan quite a few psychiatric clinicians still take phenomenological-anthropological approaches into consideration, especially when they address manic-depressive illness with typical endogenous features. This is because Shimoda's concept of “shuuchaku-kishitsu” (statothymia) has been widely accepted, together with other phenomenological views of continental origin. In the present paper the author first delineates Shimoda's concept which is based on observations of patients' personality features and the characteristics of their emotionality. He then attempts to refine this concept in spatiotemporal terms, presenting the view that in patients the past self tends to adhere to the present self (the term “shuuchaku” means “adhering to” or “preoccupied with”). He also considers that patients tend to incorporate “soto” (outer space) into “uchi” (inner space), where they believe that symbiotic relations are preserved. Finally, he argues the clinical significance of the presented views in the cultural milieu in which Japanese psychiatric practices are situated.

## 1. Introduction

About twenty years ago, DSM-III [1] was introduced to Japan and began to replace traditional diagnostic systems stemming from descriptive and phenomenological psychopathologies such as those by Jaspers [2] and Schneider [3]. However, this does not necessarily mean that every psychiatric practice now in Japan is performed on the paradigm of logical empiricism, which constitutes the mainstream of current psychiatry. As is often the case in other domains of activities in Japan, Japanese psychiatry is continuing to adopt a double standard. Most academic research follows an evidence-based method, whereas quite a few psychiatrists consider that the current results obtained by this method may fail to address insights backed by clinicians' long-term experiences. 

The tendency to adopt a double standard is especially prominent in the field of depressive illness. Although the dichotomy between endogenous/non-endogenous depressions has long been abandoned in DSM criteria, many Japanese clinicians still hold that core endogenous depression harbors specific features distinguishable from other types of depression such as neurotic depression, depressive mood manifested by people with autistic traits and so forth. One of the reasons for this tendency in Japan is that DSM criteria of major depression do not appear to delineate, either biologically or phenomenologically, a homogeneous group. This argument is also raised in Anglo-American regions. For example, Taylor and Fink [4] attempted to revive the notion of melancholia to delineate a more homogenous group of endogenous depression than the group of major depressions defined by DSM criteria. Another reason, which may be more specific to Japan, is that Japanese psychiatry has a rich tradition of phenomenological-anthropological approaches. In particular, Shimoda's notion of “shuuchaku temperament [5]” was an outstanding contribution of Japanese origin to these approaches, along with other theories of continental origin which revealed important roles of premorbid personalities and precipitating situations for the genesis of endogenous depressive illness.

To sum up, Japanese clinicians prefer a more holistic view. More concretely speaking, Japanese psychiatrists, especially those familiar with phenomenological psychiatry, emphasize in their diagnosing process not only the melancholic features of a symptomatic level as described in the DSM but also the personality traits and precipitating situations to delineate patients with endogenous features. The author addresses, in the present paper, patients with mood disorders who manifest endogenous features. 

After first introducing Shimoda's notion, the author then argues what kind of modification of this notion is required today. Finally, he underscores the significance of the presented approach, taking into consideration the cultural milieu of Japanese psychiatric practice.

## 2. The Concept of Shimoda's “Shuuchaku Temperament”

In 1941 Shimoda proposed the concept of “shuuchaku kishitsu” or “shuuchaku temperament (kishitsu = temperament).” This concept consisted of a typological description of manic-depressive patients' personalities and a hypothesis on the characteristics of their emotionality. (In the present paper the author uses the term “manic-depressive patients” to identify those with typical endogenous symptoms. It should also be stated here that the present author, together with Shimoda, does not adopt a binary position. In other words, he does not consider unipolar and bipolar disorders to be sharply distinguishable from one another.) Shimoda considered that manic-depressive patients, outside of their manic-depressive phases, are quite reliable persons in society and conscientiously fulfill obligations and responsibilities. He called this patient trait a “shuuchaku personality.” Shimoda also considered that their emotions, once aroused in relation to something, tend to persist and not easily diminish. He regarded this characteristic of their emotionality, which he called a “shuuchaku temperament,” as the neurophysiological basis of their disease. Patients with this type of personality and emotionality cannot stop overworking themselves, making every effort to preserve reliable interpersonal relationships, and so forth, and consequently fall into a depressive or manic phase. Even today the distinction of social roles between men and women is more marked in Japan than in Western countries. The overinvolvement found in a premelancholic or premanic phase in people with “shuuchaku personality” usually refers, in men, to their vocation, whereas, in women, to maintaining the order of the family or their interpersonal relations. This distinction may be becoming less marked in the current sociocultural changes in Japan. However, one of our studies [6] discloses that even in a modern Japanese enterprise women are more vulnerable to interpersonal relationships, while for men achievements in their vocation are crucial to their mood conditions.

In ordinary Japanese usage the word “shuuchaku,” originally a Buddhist term meaning “being preoccupied with” or “sticking with” something, has rather negative connotations, since Buddhism considers it indispensable to be free of preoccupations with worldly things as one tries to achieve an ideal mental state. In Shimoda's concept, however, the word “shuuchaku” merely refers to the persistence of an emotion that is neurophysiological in nature. When Shimoda's concept was introduced to Germany [7], “shuuchaku temperament” was translated into the term “statothymie” or “immobilithymie,” which means the immobility of patient emotions (stato = immobility, thymie = temperament).

## 3. Influence of the “Shuuchaku Temperament” Concept on Psychiatric Research and Practice in Japan

The concept of “shuuchaku temperament,” especially the typological description of the “shuuchaku-personality” of manic-depressive patients, was broadly accepted in Japan. Most Japanese psychiatrists agreed that those who are highly esteemed and valued in terms of their work and interpersonal reliability manifest depressive or manic symptoms after they make every effort to meet others' expectations and to fulfill their responsibilities. Shimoda's schema has both anthropological and physiological connotations. Patients' inability to meet others' expectations and fulfill their responsibilities undermines the basic ground of their self and precipitates the outbreak of the disease. Another interpretation is that their “shuuchaku temperament,” that is, the emotion which has once been aroused and does not diminish, is a persistent burden on their neurophysiological systems and results in the outbreak of not only a depressive but sometimes also a manic phase. 

**Figure 1:**
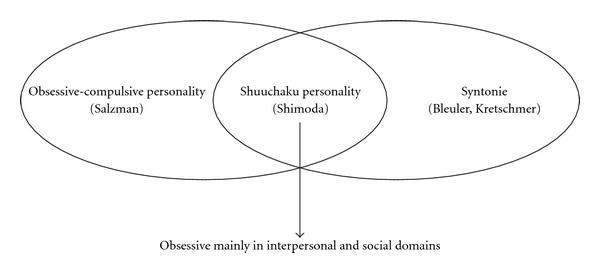
Obsessive-compulsive personality (Salzman), Shuuchaku personality (Shimoda), and Syntonie (Bleuler, Kretschmer).

 In the 1970s, Tellenbach [8], in the second edition of his book *Melancholie*, referred to Shimoda's concept and pointed out the similarity with his own notion, “Typus melancholicus (melancholic type).” His book was immediately introduced into Japan, widely accepted, and triggered vigorous discussions about the personality of manic-depressive patients, both theoretical and empirical, among researchers [9–13]. During this period the relation and the differentiation between “shuuchaku personality” and other personality traits were also investigated. The relation between “shuuchaku personality” and obsessive-compulsive personality (Salzman) [14] is of notable importance. As Kasahara [15] puts it, although “shuuchaku personality” also has an obsessive trait, its obsession is directed mainly toward social responsibilities and toward the reliability of *interpersonal* relationships. This indicates that it includes the syntonic moments. According to Bleuler [16] and Kretschmer [17], syntonic people highly value harmonious and reliable *interpersonal *relations, whereas people with typical obsessive-compulsive traits are preoccupied with *personal *domain (Figure 1). Zerssen et al. [18] elaborated on the Munich Personality Test, a questionnaire based on numerous clinical descriptions of premorbid personality traits of patients suffering from schizophrenia, unipolar and bipolar disorders, and neuroses. The MPT is composed of five dimensions, that is, extraversion, neuroticism, frustration tolerance, and rigidity and schizoidia. The dimension of rigidity is reminiscent of “shuuchaku personality,” however the MPT also does not distinguish the rigidity in interpersonal and personal domains. 

 Although neither “shuuchaku-temperament” nor “Typus melancholicus” attracted much attention in countries other than Japan and Germany, both concepts show close parallels with American psychoanalytical studies by Cohen et al. [19], Arieti [20], and Arieti and Bemporad [21], all of whom pointed out the strong concern of manic-depressive patients with social values and achievements. The main difference between these studies and the concepts of Shimoda and Tellenbach is that these psychoanalytical studies laid emphasis on the structural fragility of their patients' character that underlies their apparently desirable social adjustment, while Shimoda and Tellenbach principally focused on the aspects of their personalities, which are positively valued by society. Cohen pointed out patients' dependence on having authority manipulated into giving them approval and their lack of interpersonal sensitivity to perceive others as persons who are different from themselves. According to Arieti and Bemporad, such patients are bargaining their autonomy, which is oriented toward social achievements in order to elicit nurturance from an authoritative person, whom Arieti called “the dominant other.”

## 4. Significance and Limitations of the “Shuuchaku Temperament” Concept in Today's Psychiatric Practice in Japan

In what follows, I present an overview of the significance and the limitations of Shimoda's description of the manic-depressive personality (shuuchaku personality) and his hypothesis on the characteristics of their emotionality (shuuchaku temperament) in today's psychiatric practice.

The “shuuchaku personality” is predominant even today among those who manifest typical endogenous symptoms of a melancholic type, especially in their middle age, despite the change in Japanese society over the past several decades.

However, not all endogenous manic-depressives in Japan today belong to this personality type. Above all, quite a few patients of the younger generation who manifest distinctive endogenous symptoms do not show considerable reliability in society and are even sometimes rather maladjusted. These patients often have the bipolar II disorder according to the DSM criteria or belong to the “soft bipolar spectrum” defined by Akiskal and Mallya [22]. In exemplary cases, these patients appear satisfied with the social role which they have decided to adopt in their hypomanic phases, whereas in their depressive phases they begin to suffer from the discrepancy between their own wishes and the social role they once decided to adopt. For that reason, their mood swings make it difficult for them to establish a stable social adjustment. People with the typical “shuuchaku personality” have already established their social identity. “Overidentification” with their social role [23] characterizes them and constitutes the background of future outbreaks of manic-depressive phases, whereas there is also a group of patients whose mood changes are already manifested and these mood changes are in parallel with their unstable life course and their difficulties in establishing stable social identities. 

Their interpersonal relations are also unstable. Akiskal [24] pointed out in them the coexistence of interpersonal sensitivity and impulsive extroversion. 

With regard to Shimoda's hypothesis on the characteristics of their emotionality, this hypothesis appropriately explains the cases in which overwork precipitates the manifestation of symptoms. In such cases the patients overwork themselves not only because of their strong sense of obligation but also because of their excessive affective involvement in their work. Their inability to avoid affective involvement, which is prominent in their premelancholic phases, results in a vicious circle, from which they fall into melancholic or manic phases.

However, this hypothesis also has some limitations. One is that in some patients this characteristic of emotionality cannot be regarded as an inherent predisposition as postulated by Shimoda. Some patients claim that when they were young they were able to deal with things without persistent preoccupation. In such patients the tendency of their emotions to persist should be regarded as a trait, which did not present itself until a certain point in time in their premelancholic period.

Another limitation is that there are many people in whom overwork as a result of persistent affective involvement does not precipitate manifestations of a disease. Paradoxically, some manifest depressive symptoms after they have become free from impositions (Entlastungsdepression, Schulte [25]). Also, in other cases, in which a loss of an important person or a change in surroundings constitutes factors precipitating a melancholic phase, the persistence of the emotion is not clearly demonstrated, because these cases are not characterized by the persistent grief which would be the result of loss or change. Rather “not being able to feel grief” characterizes them (Schulte [26]). Nor, among patients in the “soft bipolar spectrum”, can persistent involvement in work be usually found to act as a precipitating factor.

## 5. Reconsidering the “Shuuchaku Temperament” Concept for an Improved Understanding of Manic-Depressive Patients in terms of Time

I would now like to reconsider the concept of “shuuchaku temperament” in order to broaden its validity. For that purpose I will focus on the term “shuuchaku” in its ordinary Japanese meaning (i.e., to stick to something) and pose the question, what is it that, the patients “stick to”? My assumption here is that, in terms of time, the patients stick to the past self, which could have been or which should have been but which was not. More precisely, the past self adheres to the present self of patients.

First, I would like to begin this reconsideration with the patients of “shuuchaku personality” or “Typus melancholicus” by referring to Tellenbach's observation of the preoccupation with precision at work in this type of patients. Tellenbach pointed out in them “the preciseness, which holds them back,” meaning that they cannot bring their work to a conclusion and are always revisiting it to achieve “preciseness.” In them the self, which should or could have achieved preciseness but has not, remains stuck in the present. This “shuuchaku” or “sticking” drives the patients to continue working. In my opinion this “sticking” to their activities underlies the “persistent emotion” which Shimoda postulated as a predispositional basis of the disease, and *not* vice versa.

This feature, which I here call “shuuchaku activity,” characterizes the premelancholic phase of this type of patient, in whom the past self sticks to the present self, driving it continuously to be involved in activities that would improve preciseness. However, in the beginning of the melancholic phase the patients finally begin to lose the potential to achieve the results and to manifest symptomatic agitations, not only because the patients burn out in their attempt to complete an enormous amount of work but also because of the intrinsic excess of their activities themselves. At that moment the bond between the past and present self is lost. The patients come to be left in the present that has lost its continuity with the past, bereft of the drive for future activities. It is at this point that they are overtaken by what Von Gebsattel [27] called “Werdenshemmung,” the impossibility of becoming.

The meaning of a loss of an important person or a change in his surroundings as a precipitating factor also becomes clear when we pay attention to this “sticking” feature (shuuchaku) lying deep inside the patient's mode of existence in terms of time. In such a patient the past self, which was at one with his or her important persons and surroundings, still sticks to the present and drives the present self to reclaim them. However, in these cases, in reality there is no possibility from the very beginning for them to reclaim them, while patients losing the preciseness of their work still have, at least in the beginning, the possibilities to reclaim preciseness through their own efforts. Thus, the loss of an important person or a change in surroundings could directly undermine the very drive they need for their activites. What the patients feel when faced with this fundamental loss of the basic ground of their activities is not grief.

Focusing on the “sticking” feature also sheds light on our understanding of the patients belonging to the “soft bipolar spectrum.” Because of their maladjustment these patients pose quite a contrast to patients with the “shuuchaku personality.” However, they often become maladjusted as a result of their tendency to seek an ideal social role. Even if they have decided to play a certain social role, they begin to entertain again the possibility of finding a better role, in the course of which they become gradually maladjusted. In them the past self, which has already made a certain decision but could have made a better one, sticks to the present self and drives it to make yet another decision.

As a whole, the “sticking” manifests in premelancholic or premanic situations as agitation, which is principally still not yet considered to constitute melancholic or hypomanic “symptoms” per se. In a working setting, it manifests as agitated overinvolvement in activities reclaiming the possibility of the ideal fulfillment of the task. When one experiences the loss of an important person or surroundings, it manifests as preoccupation with a past in which one harbored symbiotic relations with them. Even in young people who have already been afflicted with soft bipolar mood changes, it manifests as an endless indecisiveness, which makes it difficult for them to establish their social identities. 

The sticking feature can also be considered to underlie the difficulties of the manic-depressive patients in changing a plan they have set up and their failures to make themselves open to unexpected events or others' novel opinions. Generally speaking, one's activities are only to some extent determined by the plan made in the past. However, in manic-depressive patients, the past self, which has set up a plan, strongly sticks to the present self, and this sticking makes it difficult for the present self to change the plan, since to do so would break the bond between the two selves and undermine the basic ground of the activities of the present self. Therefore they tend to exclude themselves from unexpected encounters with new things and encounters with others' novel opinions. This understanding gives a clue to the well-known conservative attitude toward the life of melancholic patients [28]. It also distinguishes the meticulous preoccupation with the past in melancholic patients from the machine-like obsession of patients with autism.

## 6. “Shuuchaku Activity” and “Syntonie (Bleuler)”

This tendency of manic-depressive patients to exclude themselves from unexpected encounters especially when they are engaged in “shuuchaku activities” might seem to contradict “syntonie,” a well-known trait of manic-depressive patients. Bleuler [16] and Kretschmer [17] emphasized that, contrary to schizophrenics, manic-depressives are syntonic, meaning that they show great receptivity to others and are capable of behaving in harmony with other people. The intense involvement in work based on obsessive planning, which might lead to the exclusion of encounters with others and the great receptivity to others, which might lead to the addiction to human contacts, are two apparently major contradictory traits of manic-depressive patients.

 To understand the relation of these two traits, it is necessary to keep in mind the following two things.

 First, the exclusion of unexpected encounters of manic-depressive patients does not involve their loss of contact with other people. Even if their intense involvement in work prevents them from being open to new experiences, they are working to meet others' expectations and they are also expecting rewards from others consciously or unconsciously. However, they are not open to others who appear to be outside of the relationship they are already engaged in.

 Second, their great receptivity to others cannot be equated with openness to others. It is true that they live in harmony with other people but this is done only by attuning themselves to others or expecting others to attune themselves to them. They lack the capability of dealing with others as others who differ from them by nature, as Cohen pointed out.

 The space composed of homogeneous people, where people are easily resonant with each other, can be called “inner space,” whereas the space composed of heterogeneous people, where people must respect the differences among themselves, can be called “outer space.” The Japanese especially have a marked tendency to live in the dichotomy between inner (uchi) space and outer (soto) space, and they assume a culturally specific stance toward it. In what follows, I would like to reconsider the concept of “shuuchaku-temperament” in terms of space, by asking what kind of space it is that the patients are sticking to.

## 7. Reconsideration of the “Shuuchaku Temperament” Concept in terms of Space with Reference to the “Uchi/Soto” Dichotomy

In Japan, an “uchi-” type relation is one of intimacy in a group such as a family or a community. In an “uchi” or inner space, a person can afford to indulge himself to some extent, relying on the consciousness that “we belong together” or “we understand each other.” Members in an “uchi” space are required to help each other and share interests against the pressures from “soto” or outer space.

“Soto” or outer space indicates the outside of this “uchi” or inner space and is composed of people unknown or unfamiliar with each other. To establish a “soto-” type relationship in this space, one is required to play an appropriate social role and to follow public norms.

 The distinction between “uchi” and “soto” cannot be made based only on the concrete concept of space. For instance, parents educate a child to behave in accordance with public norms. Although this education is conducted within families or in “uchi” space, it is directed toward “soto” or outer space. It is an important task of the family to provide a child with the basis for its future behavior in “soto” space. This must be done within a family, which is principally an “uchi” or inner space.

 What is prominent in Japanese society is its strong tendency to incorporate “soto” or outer space into “uchi” or inner space. For instance, human relations in companies are principally of the “soto” type. However, in Japan they often assume a pseudofamilial character and become “uchi-” type relations. In Japan, people are not accustomed, at least at the psychological level, to deal with the conflicts in “soto” space based on definite norms and contracts. Rather they consider it desirable to deal with them based on implicit common sense, which they believe in sharing. The more “soto” or outer space is incorporated into “uchi” or inner space, the more people are pressured into conforming to an implicit collective norm shared within “uchi” or inner space. At the same time “soto,” which has not been incorporated into “uchi,” tends to become the space where due respects are not paid.

 Now let me return to manic-depressive patients and see how this “uchi/soto” dichotomy can contribute to understanding them in terms of space.

 The patients of the “shuuchaku personality” or Typus melancholicus show a prominent concern to fulfill their social roles and responsibilities in “soto” or outer space. They attempt to make every effort to avoid the situation in which they fall in debt (Tellenbach [8]) in terms of their obligations and responsibilities in “soto-” type relations. However, further scrutiny often reveals that they are preoccupied with or stuck to the “uchi” or inner space in which they believe symbiotic relations can be preserved. In the previous chapter I pointed out that in some patients the tendency of their emotion to persist does not become apparent until a certain point in their premelancholic phase. It is often the case that such patients experience some disappointment in their relations in “uchi” space at that point. For instance, after having difficulties in maintaining a symbiotic relation with their family members, they come to be intensely involved in activities in “soto” or outer space, since involvement in this type of activity at least brings them opportunities to get results and, in addition, rewards from others. It is also often the case that patients implicitly try to restore symbiotic “uchi-” type relations through their efforts, which are apparently directed toward “soto” space. The future manic-depressives among Japanese workers overextend themselves not only to fulfill their responsibilities in “soto” space, but often do so expecting emotional rewards from their colleagues. They consciously or unconsciously expect that symbiotic relations of the “uchi-” type can be preserved in their working place if they work hard enough.

 Those examples reveal the dynamics of manic-depressive patients with a “Shuuchaku personality,” who harbor a strong preoccupation with “uchi-” type relations beneath their intense concern about social obligations to establish a façade in “soto” space. I think it is also worthwhile to keep this dynamic in mind for better understanding of those patients, whose dependency appears in the foreground, rather than hidden beneath their façade as a socially reliable person. Cohen et al. [19] pointed out that these patients do not verbalize their request for others to meet their needs but rather convey it only by implication and that this attitude is interpreted by others as manipulation or coercion. I believe that these patients can convey requests only by implication since they experience their explicit request as “a debt to others” in “soto-” type relations, which they want to avoid by all means. They expect others to meet their needs in “uchi-” type relations, in which they believe explicit requests are not required.

 Manic-type patients [29] are characterized by the evasion of restricted “uchi” or inner space, which puts implicit pressure on them, by seeking the broad “soto” (outer) space. But this does not mean that they are open to “soto” space in its intrinsic sense. Rather they seek “soto” space insofar as this “soto” space can be incorporated into “uchi” space. They just try to swallow “soto” space. This is supported by the fact that the public norms in “soto” space cause them anxiety and that they rebel against them. It is also confirmed by their attack against those in “soto” space who are not willing to attune themselves to them. 

In current empirical personality studies, “neuroticism” is often pointed out as a risk factor of future depressive illness. However, in the present author's view, one should revive the serious consideration of “dependency,” a focus of attention in early psychoanalytical studies, as a core feature of manic-depressive patients. The foregoing discussion reveals that dependency can be pointed out not only in depressive patients but also in manic patients, whose ego-boundary is blurred because they fail to respect “otherness” in others. Needless to say, the pathology of ego-boundary in manic-depressive patients mentioned above is of a completely different nature from the well-known disturbance of ego-boundary in schizophrenic patients. 

The interpersonal insensitivity pointed out by Cohen and the interpersonal sensitivity mentioned by Akiskal are not contradictory. Manic-depressive patients lack interpersonal sensitivity because they have difficulties in perceiving and accepting others who are different from them. They are sensitive in interpersonal relations because they are anxious to remain attuned to others for fear that the true differences between others and themselves are revealed. What is prominent in the patients categorized in the “soft bipolar spectrum” is the coexistence of impulsive extroversion (Akiskal [24]), which is often driven by sexual libido. That libido is what motivates them to realize perfect attunement with others. However, sexual relations, in reality, reveal the profound differences between individuals. Therefore, their impulsive extroversion is usually accompanied by feelings of anxiety.

## 8. Analogy between “Shuuchaku” in terms of Time and “Shuuchaku” in terms of Space

I have so far discussed “shuuchaku” or the “sticking” of manic-depressive patients in terms of time and space independently. To conclude my reconsideration of the concept of the “shuuchaku temperament,” I would like to discuss the analogy between the two.

 The past self of the patient, which adheres to the present self, is an unobtainable self. For instance, it is the self which should have realized complete achievements, which was perfectly one with its surroundings, and which could have chosen an ideal social role. However, it is the possibility to restore this self, however illusory, that drives the present self of the patients to continue its activities. In the same way, the “uchi” space with which patients are preoccupied is an unobtainable space, since it is an illusion that symbiotic relations are preserved in “uchi” space. However, the preoccupation with “uchi” space drives the patients to continue their activities in “soto” space.

These patients cannot afford to enjoy an untroubled life when they retreat from the present to the past and from “soto” space to “uchi” space. Such retreats bring the patients only the peace of the graveyard (Friedhofsruhe, Matussek [30, 31]), which also leads them to depression. Rather it is indispensable for the patients to be forever driven to continue their activities in “soto” space to maintain their mood. Thus, becoming free from the impositions in “soto” space (Entlastung) can also precipitate depression.

## 9. The Specificity of Japanese Culture and Evaluation of Manic-Depressive Patients in Japan

Finally, I must ask whether the foregoing discussions of the manic-depressive patients, especially those in terms of space, are valid only for Japanese patients or are also valid for non-Japanese.

 Although the “uchi/soto” dichotomy may be more prominent in Japanese society than in other societies, I consider that the foregoing discussion is also valid, at least to some extent for non-Japanese patients. As a matter of fact, I referred to the descriptions by Cohen, Akiskal, and Schulte in the foregoing discussion and tried to show how these descriptions can be interpreted from the standpoint of “uchi/soto” dichotomy. I consider that what is specific is not the “uchi/soto” dichotomy itself but the tendency of Japanese culture to incorporate “soto” space into “uchi” space. Such a tendency shares something in common with the tendency of manic-depressive patients, as the foregoing discussions show. What does this tell us about the clinical view of manic-depressive patients in Japan?

 I consider that this may be why manic-depressive personalities have not been considered to be highly pathological but have often been positively regarded in Japan. In the previous chapter I referred to Cohen's patient who cannot verbalize his requests for others to meet his needs but conveys them only by implication. This kind of attitude can also be a burden to the people around him but may be more tolerated in Japanese society than in other societies, for it is a common standard of classical Japanese hospitability to anticipate others' needs before they are verbalized and to offer help. Furthermore, the attitude of “shuuchaku personality” patients who sacrifice themselves by overwork in order to restore harmony in the working place is regarded as quite positive rather than a betrayal of workers' rights in Japan.

 It could be argued that Japanese people in general and manic-depressive patients in particular share the problem, that is, a tendency to direct themselves toward “soto” space without severing symbiotic bonds in their “uchi-” type relations. I consider that because of this similarity between the tendency of Japanese in general and that of manic-depressive patients in particular, Japanese psychiatrists have been able to provide a meaningful description of the positive aspects of the manic-depressive personality. 

 Of course, this is not to say that all Japanese are manic-depressives. Among ordinary Japanese the above-mentioned tendency is counterbalanced by at least two factors; one is the religion-based trait of Japanese to consider “shuuchaku” or preoccupation with worldly things in a negative light, as mentioned at the beginning of the present article. The other is the Japanese technique of human relations known as “ashirai.” The word “ashirai” was originally used to describe the performance of “noh” music, in which a flutist accompanying a singer performs without sharing any predetermined musical codes and rhythms. In human relations, “ashirai” is the technique to show high respect to the “otherness” of others and realize smooth human relations in “soto” space, without depending on rigidly codified rules or dialectic discussions. To avoid preoccupation or “shuuchaku” together with developing the technique of “ashirai” may be of therapeutic relevance for manic-depressive patients in Japan.
